# Decreased birth weight after prenatal exposure to wildfires on the eastern coast of Korea in 2000

**DOI:** 10.4178/epih.e2023003

**Published:** 2022-12-09

**Authors:** En-Joo Jung, Ah-Young Lim, Jong-Hun Kim

**Affiliations:** 1 Department of Public Medical Center, Seoul National University Bundang Hospital, Seongnam, Korea; 2 Department of Social and Preventive Medicine, Sungkyunkwan University School of Medicine, Suwon, Korea

**Keywords:** Birth weight, Prenatal exposure, Trimester, Wildfire, Disaster, Korea

## Abstract

**OBJECTIVES:**

In April 2000, a series of wildfires occurred simultaneously in five adjacent small cities located on the eastern coast of Korea. These wildfires burned approximately 23,794 hectares of forestland over several days. We investigated the effects of prenatal exposure to the by-products generated by wildfire disasters on birth weight.

**METHODS:**

Birth weight data were obtained for 1999-2001 from the birth registration database of the Korean National Statistical Office and matched with the zip code and exposed/unexposed pregnancy week for days of the wildfires. Generalized linear models were then used to assess the associations between birth weight and exposure to wildfires after adjusting for fetal sex, gestational age, parity, maternal age, maternal education, paternal education, and average exposed atmospheric temperature.

**RESULTS:**

Compared with unexposed pregnancies before and after the wildfires, mean birth weight decreased by 41.4 g (95% confidence interval [CI], -72.4 to -10.4) after wildfire exposure during the first trimester, 23.2 g (95% CI, -59.3 to 13.0) for exposure during the second trimester, and 27.0 g (95% CI, -63.8 to 9.8) during the third trimester. In the adjusted model for infants exposed in utero during any trimester, the mean birth weight decreased by 32.5 g (95% CI, -53.2 to -11.7).

**CONCLUSIONS:**

We observed a 1% reduction in birth weight after wildfire exposure. Thus, exposure to by-products generated during a wildfire disaster during pregnancy may slow fetal growth and cause developmental delays.

## GRAPHICAL ABSTRACT

[Fig f4-epih-45-e2023003]

## INTRODUCTION

Wildfire exposure is an important and increasing risk factor for public health that is particularly relevant in Korea, where forests occupy 65% of the land and the risk of wildfires is always present [1]. From 2011 to 2020, the annual average number of wildfires in Korea was 447, and the damaged area per fire was 2.1 hectares, which was 8% and 60% lower than the corresponding values of 487 and 5.0 hectares from 2001 to 2010, respectively. However, since 2017, large-scale wildfires have continued to occur every year [2].

Forest fires are especially prevalent in the Gangwon Province of Korea. This province accounts for 22% of the country’s total forest area and predominantly comprises coniferous (31%), mixed (60%), and broad-leaved (10%) forests. Including mixed forests, more than half of the province’s area is planted with coniferous trees. Coniferous leaves are particularly vulnerable to forest fires because they contain highly volatile components [3,4]. Moreover, in Gangwon Province, cold and dry seasonal winds blow from continental China in spring. These monsoon winds cross the Taebaek mountain range and cause the Föhn phenomenon, whereby relatively high temperatures co-exist with dry winds blowing off the east coast region [5]. Indeed, the 2000 east coast wildfire started on April 7 in Goseong County in Gangwon Province; it was characterized by the simultaneous occurrence of 10 wildfires in several areas, including Gangneung, Donghae, and Samcheok. Importantly, the wildfires lasted for a total of 9 days until April 15 and damaged approximately 23,794 hectares of forest.

Wildfire smoke contains various components that worsen air quality, including fine particulate matter (PM), nitrogen dioxides, carbon monoxide, volatile organic carbon, and other metals and toxins. This worsened air quality adversely affects health [6,7]. Largescale wildfires especially generate large amounts of atmospheric emissions in a short period and typically increase PM concentrations beyond the 24-hour PM standard. This adversely affects people’s health, especially that of susceptible populations [8]. particulate matter with aerodynamic diameter < 2.5 μm (PM_2.5_) is a particular health concern: it is known to infiltrate the human body through the airway and cause respiratory and cardiovascular symptoms by inducing oxidative damage to the body and inflammatory reactions in organs [9]. The population exposure levels due to wildfires vary widely depending on the size of the burned area, speed of dispersion by wind, level of access to flame and people, and strength of the flames.

Previous research has shown that PM produced by wildfires is more toxic than that generated in urban environments [10]. Infant groups, including fetuses, and elderly populations with underlying diseases are particularly at risk of PM-induced adverse health effects [11-16]. A study published in 2022 confirmed that the medical use of residents of wildfire-affected areas in Gangwon Province increased compared to neighboring non-affected regions [17]. However, few studies have quantified the potential health impacts of exposure to wildfire smoke or prescribed fires in Korea. In this study, we investigated the relationship between exposure to wildfire smoke and the birth weight of fetuses in Korea.

## MATERIALS AND METHODS

### Data source

We obtained birth weight data from January 1, 1999 to December 31, 2001, from the birth registration database of the Korean National Statistical Office. We excluded preterm (< 37 weeks gestation), and post-term births (> 42 weeks gestation), births with a reported birth weight below 1 kg or above 6 kg, and births with multiple (two or more) fetuses. Gestational age at birth was based on the number of days since the mother’s last menstrual period (LMP).

**Figure 1.**
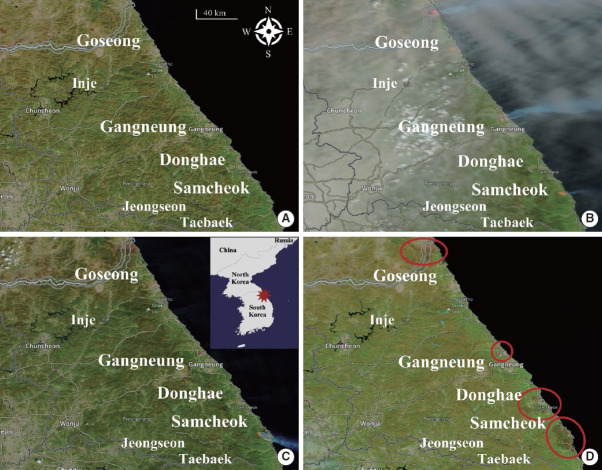
Geographic extent of the study area. Satellite photographs (A) taken the day before the fire (April 5, 2000), (B and C) at the time the fire occurred (April 6 and April 10, 2000, respectively), and (D) after the fire (April 23, 2000), with outline in red highlighting the areas affected by the fire. Source from: Corrected reflectance (bands 7-2-1) Terra/MODIS base layer; NASA Worldview (https://worldview.earthdata.nasa.gov/).

After matching the zip codes, which formed the geographic basis of this study, our sample included the Yeongdong region (coastal area), encompassing Samcheok, Donghae, and Goseong, in Gangwon Province. These areas were selected as the boundaries for the study based on satellite imagery of the fire that occurred in April 2000. By comparing the satellite photos before (A), during (B and C), and after the wildfire in the study areas (D), one can confirm the changes in the area where the wildfires occurred (Figure 1). We selected 6,921 births through these processes.

### Exposure assessment

The window of potential wildfire exposure was April 7 to April 15, 2000. Most births in the study population (n= 5,067) that were delivered before April 7 could not have been exposed *in utero*, while additional births assigned an LMP later than April 15 were classified as unexposed. All remaining births (n= 1,854) were classified as exposed based on the temporal overlap between the wildfire exposure window and gestational period.

### Covariates and statistical analysis

**Figure 2.**
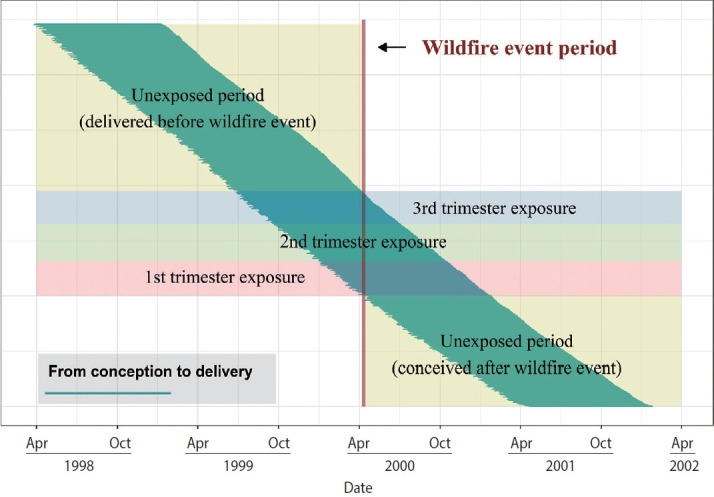
Schematic illustrating exposure assignment. Exposure status was assigned based on the overlap between the wildfire event (red) and estimated gestational intervals (horizontal segments). Dates on the x-axis correspond to the beginning of the trimesters of exposure and unexposed periods.

Because birth weight had a distribution that was close to normal, a generalized linear model (GLM) with a Gaussian distribution was used. In this model, the main exposure variable was whether exposure to wildfires occurred during gestation and gestational weeks at the time of exposure. We fit the data to a GLM with birth weight (*y_i_*) as a continuous outcome and *x_ij_* as an indicator of exposure for birth i in trimester *j*. For our primary analysis, we defined *x_i_* as a categorical variable with four levels as a dummy variable: exposed in the first trimester, exposed in the second trimester, exposed in the third trimester, or unexposed or delivered before or conceived after a wildfire event (Figure 2). We defined the first trimester as weeks 1-16 since the LMP, the second as weeks 17-28, and the third as week 29 through the end of gestation. When the wildfire event overlapped with two trimesters, we assigned exposure to the trimester with a greater number of overlapping days. We controlled for maternal and birth characteristics (*z_i_*) associated with birth weight and included terms based on the date of the LMP (*t_i_*) to control for the average exposed daily temperature (*t*).


(1) $$y_{i}=\beta_{0}+\beta_{1}x_{i1}+\beta_{2}x_{i2}+\beta_{3}x_{i3}+\eta 'z_{i}+\beta_{4}t_{i}+\varepsilon_{i}$$

In this study, an unadjusted exposure model and an adjusted model were used, and we explored the associations between birth weight and wildfire exposure to after adjusting for fetal sex, gestational age at birth, parity, maternal age, maternal education, paternal education, and exposed average atmospheric temperature in the fetus. The results of the statistical analysis were based on the effects of wildfire exposure by trimester on birth weight (g). Statistical analyses were performed using R version 4.2.0 (R Foundation for Statistical Computing, Vienna, Austria). To compare the impact with other areas that were not exposed to wildfires during the same period, we conducted a sensitivity analysis. Inje, Jeongseon, and Taebaek areas in Gangwon Province, which were not exposed to wildfires, were selected. Since these areas are located at the same latitude as the coastal areas damaged by wildfires and are located inland beyond the Taebaek mountain range, there was little chance of residents being exposed to wildfires. In the sensitivity analysis, we examined the impact of birth weight by assuming that newborns in other areas (Inje, Jeongseon, and Taebaek) were exposed or not exposed to wildfires during the same period frame as the main study, even if they were not actually affected by the wildfires (Figure 1).

### Ethics statement

This study was approved by the Institutional Review Board of Sungkyunkwan University School of Medicine (No. SKKU 2018-01-007).

## RESULTS

Of the 6,921 sampled births, 5,067 (73.2%) were unexposed during the prenatal period. Of the remaining 1,854 who were exposed, 774 (11.2%) were exposed in the first trimester, 527 (7.6%) in the second trimester, and 553 (8.0%) in the third trimester (Table 1). Most infants were delivered to mothers aged less than 35 years (93.5%), with the remaining 6.5% were delivered to mothers 35 years or older. Almost two-thirds of mothers had completed a high school education (63.2%), 29.5% had a college or higher education, and 7.3% had less than a high school education. Unlike mothers’ education, 49.5% of fathers had completed a high school education, 42.3% had a college or higher education, and 8.2% had less than a high school education. Approximately half of the mothers had one childbirth (48.0%), 41.5% had two, and the remaining 10.5% had three or more. Approximately half of the mothers gave birth at 40 weeks of gestation (49.9%), 20.3% at 39 weeks, and 16.2% at 38 weeks. Furthermore, approximately half of the exposed mothers gave birth at 40 weeks of gestation (50.9%), 21.3% at 39 weeks, and 16.4% at 38 weeks. In the first, second, and third trimesters, the average temperatures during the gestation period of the group exposed to wildfires were 15.1°C, 10.8°C, and 10.0°C, respectively. The average temperature during the gestation period in the non-exposed group was 13.0°C. We did not observe substantive differences between the exposed and unexposed groups with respect to the measured covariates, except for the average temperature (Table 1).

Both the unadjusted and adjusted estimated effects of wildfire during gestation on birth weight are reported in Table 2. Compared with unexposed pregnancies before and after the wildfire, the mean birth weight in the adjusted model decreased by 41.4 g (95% confidence interval [CI], -72.4 to -10.4) when exposed to wildfire during the first trimester, 23.2 g (95% CI, -59.3 to 13.0) during the second trimester, and 27.0 g (95% CI, -63.8 to 9.8) during the third trimester. Finally, the mean birth weight in the adjusted model for infants exposed in utero during any trimester decreased by 32.5 g (95% CI, -53.2 to -11.7) (Table 2). In the sensitivity analysis performed on newborns in Inje, Jeongseon, and Taebaek (inland regions), where wildfires did not occur during the same study period, the birth weight change was not significant (Supplementary Material 1).

## DISCUSSION

Wildfires occur sporadically and irregularly, and the probability of occurrence varies depending on weather and vegetation conditions. Surface temperature, humidity, precipitation, and wind strength greatly influence the initiation, spread, and frequency of wildfires [18,19].

**Figure 3.**
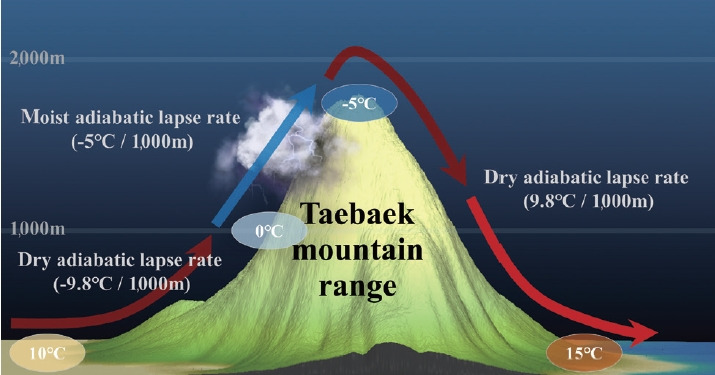
The principle of the Föhn phenomenon, which occurs when air passes through the Taebaek Mountains.

In April 2000, wildfires broke out on the eastern coast of Korea when there was high atmospheric pressure in the southern part and low pressure in the northern part of the Korean Peninsula. This was caused by a southwesterly wind in a direction perpendicular to the Taebaek mountain range (Supplementary Material 2). This arrangement of atmospheric pressure and wind direction creates strong winds from the west to the east of the Taebaek mountain range. This makes the Yeongdong region (the eastern coast of Korea) relatively dry and hot, and thus, creates conducive conditions for wildfires. This is similar to the Föhn phenomenon, which refers to the dry and warm weather that occurs downwind of a mountain range (Figure 3, Supplementary Material 3).

At the time of the wildfire in 2000, the temperature rose 3°C from 8.1°C before crossing the Taebaek Mountains to approximately 11.2-11.4°C in the Yeongdong region. Before crossing the Taebaek Mountains, weak wind speeds of less than 0.5 m/s accounted for 27.9% of the total, and most of the rest were weak southwest winds of less than 3.4 m/s. However, after crossing the Taebaek Mountains, a strong westerly wind over 8.0 m/s or more blew as it reached the coast due to the Föhn phenomenon. (Supplementary Material 2).

Wildfire smoke poses a greater risk to pregnant women than to other population groups. The greater susceptibility is due to an increase in the respiratory rate due to diaphragmatic compression from the fetus and increased blood volume. Another factor is the reduced ability to compensate for anemia that occurs in pregnant female as they must maintain the oxygen supply needed for the normal growth of the fetus [20]. While the teratogenicity of sidestream smoke has not been clearly demonstrated in humans, in experimental systems, carbon monoxide has been demonstrated to (1) decrease the metabolism of xenobiotics such as benzo-[a]-pyrene [21]; (2) interfere with metabolic and transport functions of the placenta [21]; (3) have a toxic effect on the developing nervous system of rats [21]; (4) produce minor skeletal malformations in mice and rabbits at relatively high doses [21]; and (5) at lower doses, cause a number of malformations in a dose-dependent and synergistic manner in mice deficient in protein intake during pregnancy [22,23].

Indeed, we found a birth weight loss of approximately 32.5 g for exposure during the entire period of pregnancy and a significant loss of 41.4 g for exposure during the first trimester of pregnancy. These results are in line with existing research. One study in Brazil reported that wildfire exposure during the first and third trimesters had the highest association with low birth weight [24]. In a study in Colorado, exposure to wildfire smoke PM2.5 during the first trimester was associated with decreased birth weight [25]. During the 2003 California wildfire period, the birth weight of fetuses exposed during the second and third trimesters of pregnancy decreased [26]. A recent review also indicated that maternal wildfire exposure is associated with birth weight reduction in late pregnancy [27]. This is because the embryonic period, when the formation of all major organ systems takes place, is 4-8 weeks after conception; hence, exposure to wildfire in the first trimester of pregnancy may explain the biological cause of low birth weight [28].

In general, the health impact assessment of wildfire smoke exposure in rural areas near mountainous terrain is hindered by unmeasured PM concentrations during high-concentration episodes from wildfires, called smoke waves, and limited information on population exposure to ambient PM_2.5_. Air pollution monitoring sites are concentrated around large cities and main roads, with few being installed in rural areas with low population density. An alternative can be remote sensing. One study on the analysis of major atmospheric aerosol species using Earth observation satellite data noted that major aerosol compounds (dust, carbonaceous, sea salt, and sulfate) could be detected using aerosol optical thickness, fine mode fraction, and the ultraviolet absorbing aerosol index. Those authors analyzed the California and Russian wildfires, with the aforementioned aerosols classified as carbonaceous aerosols [29]. Records for atmospheric aerosol component analysis did not exist at the time of the 2000 eastern coast wildfires in Korea; nevertheless, a large number of carbonaceous aerosols may have been generated.

According to a health impact study conducted in San Diego in 2007, wildfires have been shown to cause health problems, especially in children and elderly people who already have respiratory problems. In particular, the frequency of hospital visits tended to increase more frequently in younger children, such as infants between 0-1 years, than in the elderly [30]. A systematic literature review showed that not only children, but also pregnant females and fetuses, are vulnerable to wildfire smoke exposure. Inhaled PM interacts with the pulmonary alveolar-capillary cells and then induces an oxidative stress reaction and systematically causes an inflammatory reaction. These inflammatory reactions affect blood vessel function and increase the blood coagulation tendency. This can cause a decrease in blood flow supplying oxygen from the mother to the fetus [31]. Animal toxicology studies have shown that coarse PM causes endotoxin-induced inflammation in the lungs, while ultrafine PM affects the cardiovascular system, rather than the lungs [32].

This study has several limitations. First, because PM monitoring in this study area has been conducted since 2001, we did not perform a sensitivity analysis to distinguish between births located in areas with high and low PM levels during wildfires based on quantitative monitoring measurement data. Therefore, our results may have been mediated by air pollution, including PM. Alternatively, associations with reduced birth weight can be speculated for wildfires that occurred when atmospheric monitoring was available. Second, some unmeasured confounders may not have been completely controlled for because seasonal variation-related factors were not considered. Nevertheless, it should be noted that the examined wildfires started on April 7, 2000, and were extinguished on April 15; since it was a short period, which may perhaps reflect the real impact adjusting for the average temperature at the LMP.

Nonetheless, this study also has several strengths. First, it is the first to provide evidence on the relationship between wildfires and birth weight in Korea. Second, it underscores the necessity of creating a policy against wildfires that not only focuses on the general populace but also on specific susceptible groups, such as mothers and fetuses.

Stakeholder collaboration, including the Ministry of Environment, the Korea Disease Control and Prevention Agency, the Forest Service, the National Fire Agency, the National Disaster Management Research Institute, and local governments, is needed to assess and control the health effects of wildfires. Crucially, Korea has no guidelines for public health approaches to health impacts in the event of wildfires. As wildfire smoke is a mixture of toxic substances, including PM, appropriate guidelines are needed, including evacuation strategies, appropriate masking practices for respiratory protection, and recommendations for susceptible populations.

## Figures and Tables

**Figure f4-epih-45-e2023003:**
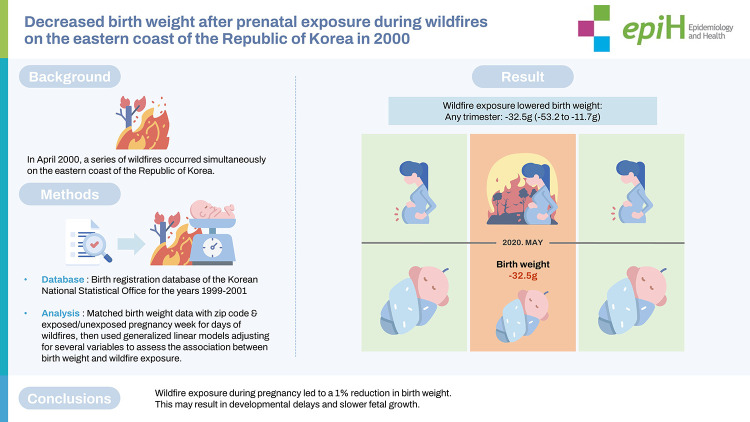


**Table 1.** Parents’ and infants’ characteristics by exposure status of wildfire and trimester of exposure

Variables		Exposed by trimester (n=1,854)			Unexposed (n=5,067)	p-value
		First (n=774)	Second (n=527)	Third (n=553)		
Fetal sex						0.571
	Male	392 (50.6)	279 (52.9)	286 (51.7)	2,695 (53.2)	
	Female	382 (49.4)	248 (47.1)	267 (48.3)	2,372 (46.8)	
Birth weight (g)		3,238±389	3,256±389	3,267±398	3,285±404	0.010
Gestational age (wk)						0.010
	37	35 (4.5)	25 (4.7)	16 (2.9)	225 (4.4)	
	38	126 (16.3)	102 (19.4)	76 (13.7)	820 (16.2)	
	39	154 (19.9)	121 (23.0)	119 (21.5)	1,009 (19.9)	
	40	403 (52.1)	240 (45.5)	301 (54.4)	2,512 (49.6)	
	41	45 (5.8)	35 (6.6)	37 (6.7)	39 (7.8)	
	42	11 (1.4)	4 (0.8)	4 (0.7)	107 (2.1)	
Parity						0.080
	1	346 (44.7)	250 (47.4)	244 (44.1)	2,485 (49.0)	
	2	338 (43.7)	223 (42.3)	256 (46.3)	2,053 (40.5)	
	≥3	90 (11.6)	54 (10.2)	53 (9.6)	529 (10.4)	
Maternal age (yr)						0.117
	<35	715 (92.4)	492 (93.4)	529 (95.7)	4,735 (93.4)	
	≥35	59 (7.6)	35 (6.6)	24 (4.3)	332 (6.6)	
Maternal education						0.496
	Less than high school	46 (5.9)	39 (7.4)	35 (6.3)	381 (7.5)	
	High school	486 (62.8)	328 (62.2)	365 (66.0)	3,196 (63.1)	
	College and more	242 (31.3)	160 (30.4)	153 (27.7)	1,490 (29.4)	
Paternal education						0.155
	Less than high school	58 (7.5)	48 (9.1)	38 (7.1)	423 (8.3)	
	High school	354 (45.7)	252 (47.8)	281 (50.8)	2,536 (50.0)	
	College and more	362 (46.8)	227 (43.1)	233 (42.1)	2,108 (41.6)	
Exposed temperature (°C)		15.1±0.9	10.8±1.2	10.0±0.7	13.0±2.2	<0.001

Values are presented as number (%) or mean±standard deviation.

**Table 2.** Estimated effect of the wildfire event during gestation on birth weight (g), by trimester

Trimester of exposure	Unadjusted model	Adjusted model^1^
Any trimester	-33.8 (-55.1, -12.5)	-32.5 (-53.2, -11.7)
First (1-16 wk)	-45.6 (-77.5, -16.9)	-41.4 (-72.4, -10.4)
Second (17-28 wk)	-27.8 (-65.7, 6.1)	-23.2 (-59.3, 13.0)
Third (≥29 wk)	-24.1 (-53.4, 16.5)	-27.0 (-63.8, 9.8)

Values are presented as effect (95% confidence interval).

1 Adjusted by fetal sex, gestational age, parity, maternal age, maternal education, paternal education, and exposed average temperature.

## SUPPLEMENTARY MATERIALS

Supplementary Material 1. Sensitivity analysis of the effect of wildfire event during gestation on birth weight (g), by trimester.

Supplementary Material 2 Meteorological phenomena and winds around the Taebaek Mountains from April 7 to 15, 2000.

Supplementary Material 3 Föhn phenomenon
