# Supplementary material for: Decreased birth weight after prenatal exposure to wildfires on the eastern coast of Korea in 2000
Source: Epidemiol Health. 2022 Dec 9;45:e2023003. doi: 10.4178/epih.e2023003 (PMC10106538; doi:10.4178/epih.e2023003)
Supplement: Supplementary Material 1. — Sensitivity analysis of the effect of wildfire event during gestation on birth weight (g), by trimester. [file epih-45-e2023003-Supplementary-1.docx]

**Supplementary materials**

**Supplementary Material 1**. Sensitivity analysis of the effect of wildfire event during gestation on birth weight (g), by trimester.

|  | Unadjusted model | | Adjusted model^1^ | |
| --- | --- | --- | --- | --- |
| Trimester of exposure | Effect (g) | 95% CI | Effect (g) | 95% CI |
| Any trimester | -10.6 | (-39.1, 17.9) | -17.0 | (-45.1, 11.1) |
| First (1-16 wk) | -11.8 | (-51.6, 27.9) | -19.6 | (-58.6, 19.5) |
| Second (17-28 wk) | -20.0 | (-68.4, 28.3) | -18.1 | (-68.4, 32.2) |
| Third (≥29 wk) | 0.8 | (-47.9, 49.5) | -11.3 | (-59.4, 36.8) |

^1^Adjusted by fetal sex, gestational age, parity, maternal age, maternal education, paternal education, and exposed average temperature.

Sensitivity analysis was performed on the birth weights of newborns (N=4,195) born in Inje, Jeongseon, and Taebaek, Gangwon Province during the same study period.
