# Supplementary material for: Decreased birth weight after prenatal exposure to wildfires on the eastern coast of Korea in 2000
Source: Epidemiol Health. 2022 Dec 9;45:e2023003. doi: 10.4178/epih.e2023003 (PMC10106538; doi:10.4178/epih.e2023003)
Supplement: Supplementary Material 2 — Meteorological phenomena and winds around the Taebaek Mountains from April 7 to 15, 2000. [file epih-45-e2023003-Supplementary-2.docx]

**Supplementary materials**

**Supplementary Material 2.** Meteorological phenomena and winds around the Taebaek Mountains from April 7 to 15, 2000.

**
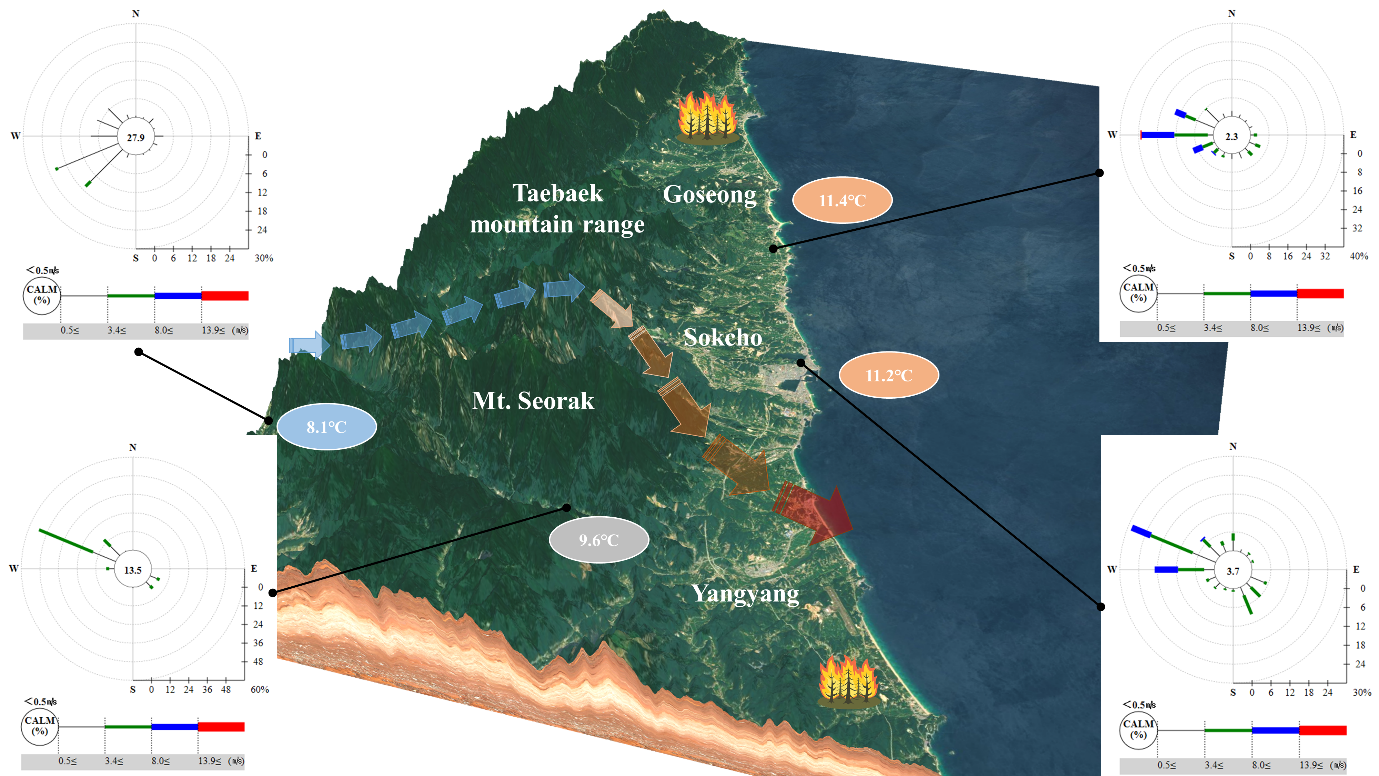
**

Source: Korea Meteorological Administration. Open MET Data Portal.

(https://data.kma.go.kr/climate/ObsValSearch/selectObsValSearchWindRose.do?)
