# Supplementary material for: Decreased birth weight after prenatal exposure to wildfires on the eastern coast of Korea in 2000
Source: Epidemiol Health. 2022 Dec 9;45:e2023003. doi: 10.4178/epih.e2023003 (PMC10106538; doi:10.4178/epih.e2023003)
Supplement: Supplementary Material 3 — Föhn phenomenon [file epih-45-e2023003-Supplementary-3.docx]

**Supplementary materials**

**Supplementary Material 3.** Föhn phenomenon

Originally, the Föhn phenomenon was described with reference to a very hot, dry wind blowing across the Alps in southern Switzerland. This is caused by variations in the temperature, which decreases (increases) depending on the degree of dryness or wetness of the air as it rises (sinks) along the ridge of a mountain. The lapse rate is the rate at which the atmospheric temperature decreases as the altitude rises in the Earth's troposphere.

When the air is dry and contains little water, this lapse rate is known as the dry adiabatic lapse rate. At this time, the rate of temperature decrease is 9.8°C/1,000 m. The presence of atmospheric water in the Earth's troposphere complicates convection processes. Water vapor contains the latent heat of vaporization. As air rises and cools, it eventually becomes saturated and cannot sustain the amount of water vapor. When this condition persists, water vapor condenses to form clouds and releases heat. The rising air before saturation follows the dry adiabatic lapse rate. However, after saturation, the rising air follows the moist adiabatic lapse rate. This rate varies widely with temperature, but a typical value is approximately 5°C/1,000 m. The reason for the difference between the dry and moist adiabatic lapse rates is that heat is released when the vapor turns into water and warms the surrounding ambient air. Theoretically, air that was 10°C before climbing the Taebaek Mountains would reach 0°C because of the dry adiabatic lapse rate at 1000 m above sea level. The water then changes to ice crystals with a wet adiabatic lapse rate of -5°C until 2000 m. As the air descends from the mountain range, the temperature rises owing to the dry adiabatic lapse rate. When it reaches the ground, the temperature rises to 15°C [33-37] (Figure 3).
